# Supplementary material for: MetaRibo-Seq measures translation in microbiomes
Source: Nat Commun. 2020 Jun 29;11:3268. doi: 10.1038/s41467-020-17081-z (PMC7324362; doi:10.1038/s41467-020-17081-z)
Supplement: Supplementary file 10 — Supplementary Data 7 [file 41467_2020_17081_MOESM10_ESM.zip › File2/Confidence_High_Taxonomy/335168_out.krona.html]

Javascript must be enabled to view this page.

members
magnitude
magnitudeUnassigned
count
unassigned
taxon
rank

335168\_out

20

2
superkingdom
20

20
976
phylum

200643
class
20

order
171549
20

20
815
family

20
genus
816

species
817
19

SRS015190\_contig\_number\_2565SRS016267\_contig\_number\_8346SRS017433\_contig\_number\_14145SRS018888\_contig\_number\_8175SRS019601\_contig\_number\_2662SRS019787\_contig\_number\_12464SRS043411\_contig\_number\_11251SRS055966\_contig\_number\_contig-100\_723.29313SRS058723\_contig\_number\_1549SRS063127\_contig\_number\_3199SRS075398\_contig\_number\_1108SRS077335\_contig\_number\_8189SRS077589\_contig\_number\_2509SRS104036\_contig\_number\_7304SRS104636\_contig\_number\_24074SRS1055022\_contig\_number\_contig-100\_947.123374SRS144362\_contig\_number\_30746SRS147139\_contig\_number\_10752SRS893279\_contig\_number\_17977

357276
species

SRS017622\_contig\_number\_176
1
